# Is the Gut Microbiome Implicated in the Excess Risk of Hypertension Associated with Obstructive Sleep Apnea? A Contemporary Review

**DOI:** 10.3390/antiox12040866

**Published:** 2023-04-03

**Authors:** Sanah S. Munir, Fatima H. Sert Kuniyoshi, Prachi Singh, Naima Covassin

**Affiliations:** 1 Department of Cardiovascular Medicine, Mayo Clinic Rochester, Rochester, MN 55905, USA; 2 ResMed Science Center, San Diego, CA 92123, USA; 3 Pennington Biomedical Research Center, Baton Rouge, LA 70808, USA

**Keywords:** cardiovascular disease, gut dysbiosis, gut microbiome, hypertension, hypoxia, obstructive sleep apnea, short-chain fatty acids, sleep fragmentation

## Abstract

Obstructive sleep apnea (OSA) is a highly prevalent sleep disorder and an established risk factor for cardiovascular diseases, including hypertension. The pathogenesis of elevated blood pressure (BP) in OSA is multifactorial, including sympathetic overdrive, vascular aberrations, oxidative stress, inflammation, and metabolic dysregulation. Among the mechanisms potentially involved in OSA-induced hypertension, the role of the gut microbiome is gaining increasing attention. Perturbations in the diversity, composition, and function of the gut microbiota have been causally linked to numerous disorders, and robust evidence has identified gut dysbiosis as a determinant of BP elevation in various populations. In this brief review, we summarize the current body of literature on the implications of altered gut microbiota for hypertension risk in OSA. Data from both preclinical models of OSA and patient populations are presented, and potential mechanistic pathways are highlighted, along with therapeutic considerations. Available evidence suggests that gut dysbiosis may promote the development of hypertension in OSA and may thus be a target for interventions aimed at attenuating the adverse consequences of OSA in relation to cardiovascular risk.

## 1. Introduction

Obstructive sleep apnea (OSA) is a sleep-related disorder marked by repetitive episodes of upper airway collapse and complete (apnea) or partial (hypopnea) cessation of breathing, causing intermittent hypoxemia, hypercapnia, sleep fragmentation and daytime consequences. Globally, it is estimated that 425 million adults aged 30–69 years suffer from moderate to severe OSA [1], with a higher prevalence among men and middle-aged and older adults [2]. The cascade of ventilatory, mechanical, hemodynamic, and endocrine responses activated by disordered breathing events leads to sympathetic nervous system activation, oxidative stress, and systemic inflammation, which, in turn, result in structural and functional impairments in the cardiovascular (CV) system. Accordingly, OSA predisposes to the development of CV diseases, including atrial fibrillation, stroke, coronary artery disease, and sudden cardiac death [3]. Among these sequelae, the relationship between OSA and hypertension is well established. Hypertension is the leading risk factor for morbidity and mortality worldwide and increases the risk of severe CV complications [4]. While it is known that the pathogenesis of hypertension in OSA involves the above-mentioned mechanisms, recent, intriguing evidence also favors a role for the gut microbiota in OSA-related hypertension. The microbiota is the assemblage of microorganisms, including bacteria, archaea, viruses and fungi, which inhabit an environment such as the gastrointestinal tract. The number of microorganisms populating the human gut is 10 times higher than the total number of cells in the body, and *Bacteroidetes, Firmicutes,* and *Actinobacteria* are the predominant phyla, representing 90% of the intestinal microbiota [5].

A growing body of research suggests that the gut microbial communities and their genomes (microbiome) play a critical role in the maintenance of whole-body homeostasis. By interacting with the host via metabolic, neural, and immune pathways, the microbiome contributes to a plethora of biological functions, modulating nutrient absorption, gastrointestinal motility, immune response, and lipid metabolism, to name a few. Dysbiosis of the gut microbiome, reflecting a disequilibrium in the composition and function of the microbiota, has been associated with numerous medical and psychiatric disorders [6,7], including hypertension. Emerging evidence suggests that OSA affects the gut microbial environment and that altered intestinal microbiota may contribute to the heightened risk of hypertension associated with OSA. In this review, we discuss current data on the implications of the gut microbiome for OSA-induced hypertension, presenting findings from animal models and human studies. Therapeutic considerations are also discussed.

## 2. Effects of OSA on Blood Pressure

It is well established that OSA is an independent precursor of high blood pressure (BP), with this sleep disorder being recognized as a prominent risk factor for hypertension in clinical guidelines [8,9]. Population studies indicate that 30% to 70% of adults with OSA manifest systemic hypertension, and the risk of prevalent high BP increases as OSA severity worsens [10,11,12,13]. In a pooled analysis of more than 6900 individuals, every 10-unit increase in the apnea–hypopnea index (AHI, the chief indicator of OSA severity) was found to be associated with 17% higher likelihood of incident hypertension [14]. As it pertains to hypertension profiles, the relationship between OSA and resistant hypertension is especially well documented, with OSA estimated to be present in up to 90% of patients with resistant hypertension [15,16,17]. Ambulatory BP studies show that the prevalence of isolated nocturnal hypertension is higher among individuals with OSA compared to non-apneics, and the physiologic fall in BP which occurs at nighttime (namely nocturnal dipping) is attenuated or even reversed in OSA [18,19]. In more recent times, distinct OSA subtypes have emerged as particularly hazardous and independently associated with adverse outcomes, including hypertension. Rapid-eye movement (REM)-related OSA is a stronger predictor of hypertension than non-REM OSA [20], possibly because of the higher degree of instability in CV activity manifested in REM sleep and/or the greater reactivity to disordered breathing events observed in this stage. The OSA phenotypes characterized by high hypoxic burden [21], short sleep duration [22], or excessive daytime sleepiness [23] have also been linked to greater vulnerability to elevated BP.

**Figure 1:**
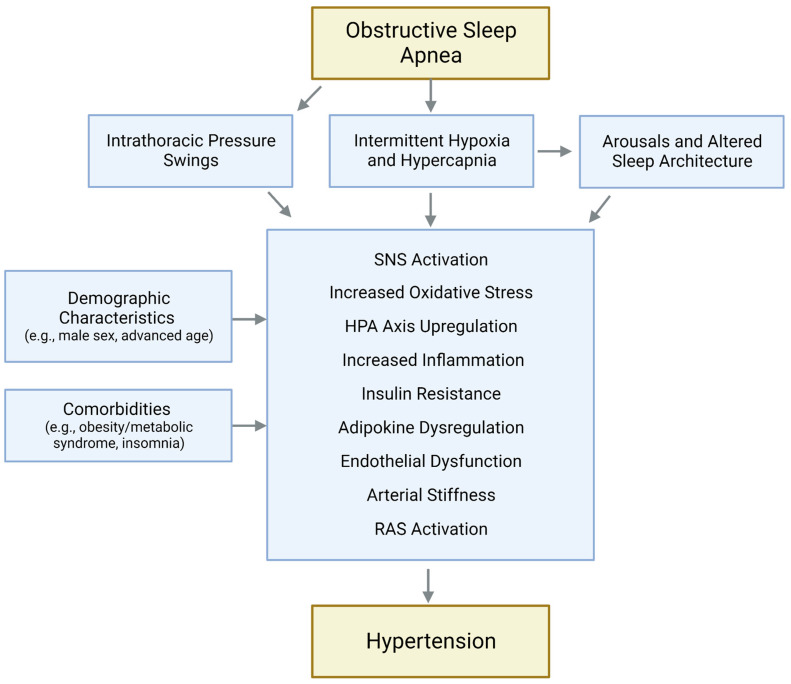
Pathophysiology of hypertension in obstructive sleep apnea. HPA—hypothalamic–pituitary–adrenal; RAS—renin–angiotensin system; SNS—sympathetic nervous system. Created with BioRender.com.

The pathophysiology of OSA-induced hypertension involves multiple determinants and biological pathways activated by disordered breathing events (Figure 1). Obstructive respiratory events during sleep trigger a cluster of physiological mechanisms that culminate in acute BP elevations. Hypoxemia and hypercapnia secondary to reduced or interrupted airflow stimulate peripheral and central chemoreceptors, leading to increased sympathetic outflow, which is further promoted by the lack of sympatho-inhibitory effects of respiration. Consequently, BP begins to rise, and the pressor response peaks upon resumption of breathing, owed to the abrupt increase in cardiac output in the setting of continued peripheral vasoconstriction. Arousal-mediated sympathetic stimulation, increased intrathoracic pressure, and vagal withdrawal following restored ventilation further contribute to accentuate the acute post-apnea BP surge. Over time, prolonged exposure to recurrent episodes of airway obstruction and related pressor responses results in sustained BP increases. In addition, enhanced chemosensitivity and sympathetic overdrive also manifest during wakefulness, fostering a transition towards established hypertension. Upregulation of the hypothalamic–pituitary–adrenal axis, impaired endothelial function, increased arterial stiffness, renin–angiotensin system stimulation, inflammation, and metabolic derangements are also evident in OSA and are implicated in the development of overt hypertension and future CV disease in this population [24,25,26]. The heightened risk of hypertension exhibited by OSA patients is compounded by concurrent demographic and clinical risk factors associated with both elevated BP and OSA, including advanced age, male sex, obesity, type 2 diabetes, and comorbid sleep disorders.

## 3. Effects of Gut Dysbiosis on Blood Pressure

**Figure 2:**
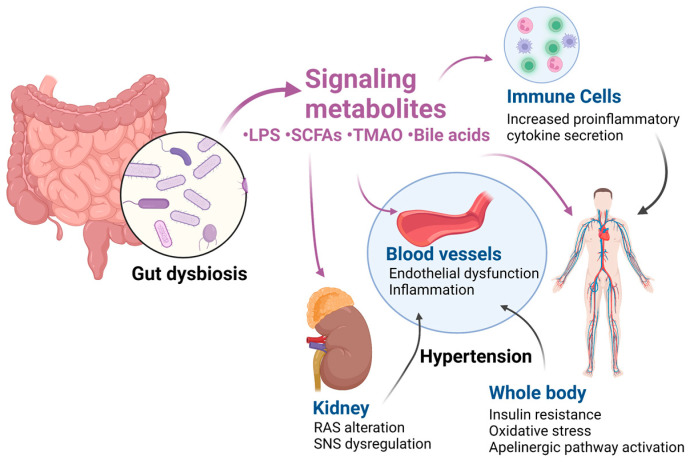
Proposed pathway through which perturbations in the gut microbiome may cause hypertension via altered signaling metabolites. LPS—lipopolysaccharide; RAS—renin–angiotensin system; SCFA—short-chain fatty acids; SNS—sympathetic nervous system; TMAO—trimethylamine N-oxide. Created with BioRender.com.

Accumulating evidence identifies gut dysbiosis as a key factor contributing to BP dysregulation and to the development of hypertension (Figure 2).

Case–control studies comparing normotensive and hypertensive individuals show taxonomic and functional differences between groups, with lower intestinal microbial diversity and richness in those with hypertension [27,28,29]. Distinct microbial signatures are evident in relation to high BP, with greater abundance of potential pathogenic taxa, including *Prevotella, Klebsiella*, and *Streptococcus*, alongside a reduction in health-promoting species such as *Bacteroidetes* bacteria which produce short-chain fatty acids (SCFAs), including *Roseburia* and *Faecalibacterium* genera of the *Lachnospiraceae* and *Ruminococcaceae* families [28,29,30,31].

Epidemiological investigations support these observations obtained from relatively small samples, with measures of microbial diversity and specific taxa, including members of the *Ruminococcaceae, Clostridium*, *Lactobacillus,* and *Roseburia* species, which are independently associated with BP levels and hypertension diagnosis in large, diverse populations [32,33].

Animal models of hypertension exhibit less diverse and rich microbiota than normotensive animals [27], together with an increased *Firmicutes* to *Bacteroidetes* (F/B) ratio, which is a conventional marker of gut dysbiosis. At the genus level, depletion in butyrate-producing bacteria such as *Coprococcus* and *Pseudobutyrivibio* and an increase in lactate producers such as *Streptococcus* and *Turicibacter* appear to be the main contributors to the imbalanced F/B ratio in animals with hypertension [27]. A causative role of disrupted gut microbiome in the pathogenesis of hypertension is further demonstrated by experiments in mice and rats showing significant increases in BP after transplantation of cecal contents from hypertensive donors [28,34].

A complex network of mechanisms mediates the hypertensive effects of disrupted microbiome.

Gut dysbiosis may increase intestinal permeability and, along with microbial translocation, lead to increased levels of circulating lipopolysaccharide (LPS), a marker of endotoxemia [35]. LPS are components of Gram-negative bacteria such as *Escherichia coli*. By stimulating macrophages and monocytes, these bacterial toxins facilitate the onset of low-grade systemic inflammation, with consequent cardiometabolic consequences [36]. LPS-induced inflammation is evident in the vasculature, and LPSs impair endothelial function [37]. Elevated levels of LPS are also associated with high BP [31].

As a byproduct of bacterial fermentation of dietary fibers in the gut, SCFAs such as butyrate, acetate and propionate regulate microbial health by maintaining gut integrity and immune system homeostasis. SCFAs stimulate mucus synthesis and inhibit bacterial translocation, while suppressing neutrophil, macrophage, and pro-inflammatory cytokine activity. SCFA-producing bacteria such as *Ruminococcaceae* and *Lachnospiraeceae* families facilitate the proliferation and differentiation of T-regulatory (Treg) cells and decreases in T helper17 (Th17) cells, thus affecting immunity [38,39,40]. Furthermore, by binding to G-protein-coupled receptors in the vasculature and kidneys, SCFAs regulate the release of renin, sympathetic nervous system activity, and arterial vasodilation [41]. In addition, SCFAs are involved in the regulation of insulin sensitivity and oxidative stress [42,43], both of which contribute to BP modulation.

As lactate has been associated with heightened hypertension risk [44,45], increased relative abundance of lactate-producing bacteria such as *Streptococcus* and *Lactococcus* may contribute to BP elevation.

A choline metabolite, trimethylamine N-oxide (TMAO), has been linked to increased CV risk due to its pro-inflammatory and pro-atherogenic characteristics [46,47]. TMAO may also directly affect BP regulation via stimulation of angiotensin II-mediated vasoconstriction [48].

In addition, the relationship between intestinal microbiota and hypertension may be modulated by diet composition. Salt-sensitive models develop hypertension after receiving cecal content from salt-resistant rats [49]. A high-sodium diet affects the intestinal microbiome by reducing *Lactobacillus*, increasing T-helper 17 cells and ultimately raising BP in a murine model as well as in humans [50].

## 4. Altered Gut Microbiome in OSA: Implications for Hypertension Risk

The impact of OSA on the gut microbiome has been receiving increasing attention, with accumulating research showing perturbations in diversity, composition, and function of the intestinal microbiota in those with OSA. Given the vast ramifications of gut dysbiosis for the host’s metabolism and health, it is conceivable that microbiota derangements may contribute to the exacerbated vulnerability to hypertension manifest in this population.

### 4.1. Clinical Studies

Wang et al. [51] found that OSA patients had lower diversity and higher F/B ratio than controls without OSA, with increased *Firmicutes* and decreased *Bacteroidetes*, along with higher abundance of members of the *Lachnospiraceae, Veillonellaceae* and *Enterobacteriaceae* families and decreased *Rikenellaceae* families. At the genus level, *Clostridium_XIV* was enriched while *Alistipes* was reduced in those with OSA compared to healthy controls. As *Rikenellaceae* and *Alistipes* are SCFA producers, their depletion may promote inflammation in OSA. Consistent with a shift towards a pro-inflammatory state in OSA, in this study, the Th17/Treg cell ratio was also decreased in apneic patients. Despite individuals with and without OSA showing a similar F/B ratio in another case–control study, gut microbial taxa features were found to accurately identify those with OSA in a receiver operating characteristic analysis study [52]. Several genera differ between OSA severity groups, including altered abundances of *Faecalibacterium*, *Oscillobacter*, *Megamonas, Ruminomococcaceae, Alistipes, Bifidobacterium, Gammaproteobacteria,* and *Micrococcus*. In line with prior observations, the underrepresentation of bacteria producing SCFAs, such as *Faecalibacterium,* and the overgrowth of pathogens fostering production of LPS, such as *Gammaproteobacteria,* was accompanied by and potentially causally related to higher levels of inflammation in severe OSA, presumably via a damaged gut barrier. In addition, functional analysis revealed derangements in multiple metabolic pathways, including downregulation of amino acid metabolism and insulin signaling pathway, consistent with metabolic derangements evident in OSA. By clustering genus compositions, the same group identified three enterotypes in patients with OSA, namely *Bacteroides*, *Ruminococcus,* and *Prevotella* [53]. Polysomnographic data showed greater sleep disruption in patients with the *Prevotella* enterotype, although circulating inflammatory markers did not differ from non-OSA.

The association between OSA severity and aberrant microbiota was replicated in a recent study [54]. Severe OSA patients exhibited higher relative abundance of *Fusobacterium*, *Megamonas*, and *Lachnospiraceae* and lower *Anaerostipes*. Notably, *Fusobacterium* has pro-inflammatory properties and has been associated with CV disease [55], as has the LPS-promoting bacterium *Megamonas* [56]. Network analysis showed links between OSA, gut dysbiosis and intestinal barrier injury. This finding, coupled with the associations between the genera prevalent in OSA and circulating levels of lipids, glucose, and inflammatory markers, further substantiates broad repercussions of disrupted microbiota in OSA-related cardiometabolic abnormalities and, potentially, hypertension.

However, few clinical studies specifically targeted the interplay between OSA, microbiome and hypertension. Bikov et al. [57] noted a relationship between microbiota signatures of OSA and surrogate indicators of CV risk. Specifically, the relative abundance of LPS-producing *Proteobacteria,* including *Gammaproteobacteria,* was associated with measures of OSA severity as well as with hypertension. In addition to inflammation, richness of *Proteobacteria* has been linked to metabolic syndrome components, especially high BP, in obese individuals [58].

A small study found a higher F/B ratio in individuals with OSA compared to those without OSA, irrespective of comorbid hypertension [59]. Nevertheless, patients with both OSA and hypertension had lower *Ruminococcaceae* and *Lachnospiraceae* than those with OSA only, suggesting that both disorders may exert synergistic, deteriorating effects on the intestinal microbiota. Conversely, comparing hypertensive patients with and without OSA, Lu et al. [60] found that those suffering from hypertension and severe OSA exhibited greater gut dysbiosis, as indicated by lower microbial diversity and higher F/B ratio. At the genus level, in line with prior studies, lower abundance of bacteria-producing SCFAs (i.e., *Bacteroides* and *Prevotella*) was evident, while hypertensive patients with OSA had enriched *Megamonas* and *Lactobacillus* and depleted *Alistipes, Ruminococcus*, and *Fusobacteria*. Although Ko et al. [61] did not observe differences in the F/B ratio when comparing three groups of non-OSA, normotensive OSA, and hypertensive OSA patients, they found significant alterations in gut microbial taxa in the latter group of patients. While the relative abundance of SCFA-producing *Gemmiger, Dialister,* and *Oscillabacter* genera was lower in both OSA groups relative to controls, specific differences emerged when factoring in the hypertension status, as those with concurrent OSA and hypertension manifested lower abundance of *Parabacteroides* and *Akkermansia* than non-OSA. Furthermore, *Clostridium XIVa* was reduced and *Prevotellaceae* and *Bifidobacterium* genera were higher in hypertensive OSA patients compared to their normotensive counterparts. Peculiarities in the microbiotal makeup were paralleled by functional perturbations, including downregulation of arginine, proline, and linoleic acid metabolism in those with comorbid OSA and hypertension relative to non-hypertensive OSA patients. Arginine modulates the release of nitric oxide, a potent vasodilator which is suppressed in hypertension. Because the mTOR signaling pathway, which is critically involved in BP regulation via control of oxidative stress, immune response and metabolism [62], was enriched in patients with OSA and hypertension compared to OSA without hypertension, the authors postulate a role of this pathway in OSA-related hypertension.

The fecal metabolome provides a functional readout of the gut microbiome and its interactions with the host [63]. Accordingly, fecal metabolomic features accurately discriminate between OSA patients and healthy controls and are associated with OSA severity [64].

### 4.2. Preclinical Studies

Intermittent hypoxia, the hallmark of OSA, is applied in animal models to mimic human sleep disorder. The chronic intermittent hypoxia model simulates the cyclic de-oxygenation/re-oxygenation pattern characteristic of OSA, with oxygenation levels in the gut oscillating in phase with arterial hypoxemia events. Since epithelial functions, especially barrier and absorptive functions, are modulated by oxygen, hypoxia can impact the gut microbiome. Repeated cycles of hypoxia and re-oxygenation may favor the growth of anaerobic bacteria in the gut and injure the epithelium, leading to gut dysbiosis, increased permeability of the intestinal mucosa, bacterial translocation, and thus compromised intestinal function. Changes in metabolites, especially SCFA and bile acids, may also ensue and can contribute to the deterioration of intestinal integrity, increased inflammation and end-organ damage, thereby promoting the development of OSA-related hypertension. In addition, as discussed above, alterations in the renin–angiotensin system secondary to decreased SCFA and impaired arginine metabolism may be involved.

Accordingly, exposure to intermittent hypoxia (IH) vs. room air elicits profound perturbations in the gut microbiome in murine models. Enhanced bacterial diversity has been observed [65], along with a shift in composition. Several members of the Gram-negative *Bacteroidetes* and *Proteobacteria* phyla are enriched, while Gram-positive *Firmicutes* are depleted in animals exposed to IH [65,66,67]. Notably, these data may be at variance with those from human studies, showing, for the most part, decreased diversity and an increased F/B ratio in OSA. While the reasons underlying such discrepancy are not completely understood, it can be speculated that the observed changes in microbiome richness due to chronic IH may be an adaptive response [59]. Metabolomic profiling shows differences in numerous compounds, particularly decreased metabolism of fatty acids and bile acids in mice undergoing IH [66,67]. IH-induced alterations in the gut microbiome and metabolome caused damage to the intestinal epithelial barrier, increasing gut permeability in these animals. Reduced expression of intestinal tight junction proteins due to IH may also be a contributory factor [68]. To better resemble the cycles of de-oxygenation/re-oxygenation, hypercapnia can be combined with IH. Models exposed to both hypoxia and hypercapnia developed profound changes in the composition of the gut microbiota, particularly in *Clostridiaceae* and *Lachnospiraceae* families and *Oscillospira* genus–taxonomic groups linked to inflammation and metabolic regulation [69,70]. This was coupled with perturbations in the metabolome, which affected fatty acids and bile acids in particular [71]. Distinct gut microbiota and metabolomic signatures of hypoxia and hypercapnia were found in murine models [72]. Nevertheless, exposure to hypoxia caused greater disruption than hypercapnia compared to room air. Notably, in fecal transplant studies, IH-induced abnormalities in gut microbiota were also found to be causally related to increased BP and were revealed as potential contributors to hypertension, including vascular dysfunction and systemic and adipose tissue insulin resistance [66,73]. IH also adversely affects the apelinergic pathway [74]. Apelin, a peptide involved in fatty acid oxidation and BP regulation [75,76], has been linked to gut microbiotal composition [77].

Another potential pathway implicated in the cardiometabolic abnormalities associated with OSA and gut dysbiosis involves circadian disruption. Mice exposed to both IH and hypercapnia exhibit altered diurnal rhythmicity of the gut microbiome and metabolome [78], increased abundance of pro-inflammatory and pro-atherogenic taxonomic members and reduced protective bacteria, with potential implications for whole-body metabolism and CV risk.

Sleep fragmentation, another cardinal feature of OSA, may also be implicated. Animals subjected to recurrent arousals from sleep manifest gut dysbiosis, with reduced diversity [79,80] and alterations in the taxonomic makeup which lead to increased intestinal permeability, LPS production and inflammation. This includes an increased F/B ratio, an overgrowth of the families *Ruminococcaceae* and *Lachnospiraceae,* and a reduction in *Lactobacillaceae* families [79]. Increased *Faecalibaculum* and *Muribaculum* and decreased *Lactococcus* and *Lachnoclostridium* genera have also been noted [80], similar to the findings in IH models. A dose–response in the relationship between sleep disruption and gut dysbiosis has been reported, with the degree of perturbation in the microbiota increasing with prolonged duration of sleep fragmentation [81]. Downstream effects of microbial alterations elicited by sleep fragmentation include systemic and adipose tissue inflammation and insulin resistance [79], presumably mediated by enhanced gut epithelial permeability; these may also contribute to BP dysregulation.

The brain–gut axis may be a potential mediator in the interplay between OSA, hypertension, and gut dysbiosis [82]. OSA may affect the gut microbiome via disrupted sleep architecture, and gut dysbiosis may compromise sleep patterns via altered metabolites, in a cyclic fashion. In keeping with the brain–gut–microbiota axis concept and its bidirectional signaling, aberrant OSA-induced microbiota promotes sleep disturbances. Transfer of cecal contents from mice exposed to IH not only caused taxonomic perturbations in the gut of transplant recipients consistent with IH effects, but also increased sleep duration and the frequency of sleep episodes, implying increased sleepiness [83]. This suggests that hypoxia-mediated alterations in microbiome may exacerbate sleep abnormalities in OSA, perpetuating its adverse health consequences. Whether this precipitates hypertension risk is unknown.

Gut dysbiosis in OSA may also result from or be potentiated by concurrent comorbidities and/or unhealthy behaviors, including sedentary lifestyle and poor diet, rather than ensuing sorely from the direct effects of disordered breathing events. This is likely to impact the likelihood of OSA-induced hypertension. This hypothesis was tested in a seminal paper by Durgan et al. [84]. The authors simulated OSA in rats through intermittent inflation of tracheal balloons during sleep and compared OSA models with sham rats under a normal chow diet or a high-fat diet condition. On normal chow diet, neither OSA nor the high-fat diet alone affected BP, while increases occurred in OSA animals fed a high-fat diet. When considering the impact on the gut microbiome, the high-fat diet increased the F/B ratio in both non-OSA and OSA models, though to a lower degree in the latter group, and no effects of OSA were seen in terms of microbiota diversity. Butyrate-producing bacteria were depleted in OSA rats fed a high-fat diet vs. normal chow, while lactate-producing bacteria increased. Cecal contents transplants to sham animals on normal chow confirmed a hypertensive effect of gut dysbiosis. Normotensive OSA recipients on normal chow developed hypertension only when receiving fecal content from hypertensive OSA donors fed the high-fat diet but not from sham models on high fat. In addition, it is worth noting that BP was unaltered in sham recipients when the animals remained on air room after transplantation. The authors concluded that while gut dysbiosis contributes to hypertension, it does not suffice as an isolated trigger, and the hypoxic stimulus is still required in order to produce sizable increments in BP.

Conversely, a synergistic effect of OSA and high-fat diet was noted by Wang et al. [85] Both IH and sleep fragmentation OSA models significantly affected intestinal taxa composition and metabolites in mice fed a normal chow and in those eating a high-fat diet. However, the combination of high-fat diet and sleep fragmentation was more detrimental, causing selective disruptions in microbial communities. Excess dietary sodium intake potentiated the effects of IH on the gut microbiota of rat models of OSA, leading to increased F/B ratio and decreased *Lactobacillus*, increased TMAO and pro-inflammatory Th1-related cytokines, and decreased anti-inflammatory cytokines [86]. This pattern was evident when considering changes in BP, with IH and a high-salt diet causing a much more severe BP elevation than either stimulus alone.

## 5. Therapeutic Considerations

Continuous positive airway pressure (CPAP) is the recommended therapy for OSA. By preserving the patency of the upper airway during sleep, CPAP abrogates apneic episodes, restoring normal sleep and alleviating daytime sleep symptoms. Although the acute physiological consequences of OSA are resolved through the elimination of disordered breathing events, including transient, apnea-induced nocturnal BP surges, the long-term CV health benefits conferred by CPAP are a matter of debate [87]. Importantly, inadequate compliance is a well-known concern with this treatment and is implicated in its suboptimal effectiveness. As it pertains to hypertension, CPAP shows modest yet significant BP-lowering effects in OSA patients, with average decreases of 2 to 4 mmHg [88,89]. The antihypertensive impact of OSA therapy appears moderately larger in patients with resistant hypertension, in whom BP falls by 4 to 5 mmHg [90]. It should be noted that considerable heterogeneity exists in the BP response to CPAP treatment, including OSA severity, hypertension phenotype, and, as mentioned above, adherence. Recent data suggest that gut dysbiosis may also play a role in the impact of CPAP on OSA-induced hypertension.

Data from animal models point toward a resistance to reversal of hypoxia-induced perturbations in the gut microbiota, despite reinstatement of normal oxygenation. Murine models previously exposed to IH or normoxic air underwent a 6-week period of normoxia, mimicking normal oxygen levels that would occur with CPAP usage. Although normoxic recovery restored normal diversity and richness of gut microbiota in OSA models, mice exposed to IH and normoxia continued to significantly segregate, even in the recovery phase [91]. Increased abundance of *Firmicutes* and decreased *Bacteroidetes* phyla were noted, and circulating levels of LPS were significantly elevated in OSA animals after normoxic recovery. Additionally, the abundance of *Lactobacillus* and *Ruminococcus* inversely correlated with LPS, while positive correlations were seen with *Mucispirillum* and *Desulfovibrio,* consistent with a relation between persistent endotoxemia and altered microbiota. Whether the remnant effects of IH on the gut microbiota, despite removal of the hypoxic stimulus, are irreversible, require longer recovery periods, or are responsive to alternative or adjunct therapeutic intervention remains to be determined.

To this end, nutritional therapeutic approaches have been shown to yield positive effects on the gut microbiome and confer CV advantages, including BP control [92]. In a meta-analysis, probiotics supplementation has been found to significantly lower BP, albeit to a modest degree—2 to 4 mmHg [93]. It is worth noting that this magnitude of decrease is comparable to that evoked by CPAP therapy. In animals, administration of *Lactobacillus murinus* prevents the onset of salt-sensitive hypertension via modulation of Th17 cells, consistent with the role of the immune system in the health-promoting impact of probiotics [50]. *Bifidobacterium breve* and *Lactobacillus fermentum* prevented BP increases and gut dysbiosis in spontaneously hypertensive rats by reducing LPS and rebalancing Th17/Treg [94].

Cardioprotective effects of probiotics have also been reported in murine models of OSA. Treatment with *Lactobacillus rhamnosus GG* prevents increases in BP in rat models of OSA-induced hypertension by lowering TMAO and rebalancing Th1/Th2 cytokines and by modulating the PI3K/Akt/mTOR signaling pathway [86]. *Lactobacillus rhamnosus GG* prevents myocardial injury in obese mice exposed to IH via activation of antioxidant pathways [95], while *Clostridium butyricum* reverses high BP in a rat model of OSA by replenishing acetate [96]. Administration of *Clostridium butyricum* also decreases gut dysbiosis and prevents epithelial inflammation in these animals. However, a recently published meta-analysis suggests that the antihypertensive effect of probiotic supplementation may not be evident in the long-term [97]. Further investigation is necessary to determine the short-term and long-term effects of probiotic use on BP, especially among OSA patients.

The use of antibiotics to modify the gut microbiome and their implications on BP regulation has been investigated. Oral antibiotics prevented OSA-induced hypertension in mice fed a high-fat diet [84] and increased the relative abundance of *Bacteriodetes,* despite reducing the overall gut biomass. Hypertensive rats treated with minocycline, an anti-inflammatory antibiotic, manifest normalized gut microbial diversity, a reduced F/B ratio, and decreased BP [27]. Minocycline increased the abundance of acetate- and butyrate-producing bacteria, while depleting lactate-producing bacteria populations.

Accordingly, administration of SCFAs may also be beneficial for BP control. Genetic hypertension rat models receiving oral butyrate or acetate do not develop hypertension, nor increases in the F/B ratio [94]. Direct acetate infusion into the cecum inhibited inflammation and BP elevation in a rat model of OSA [96]. Diets enriched with SCFAs have been found to have a positive impact on CV health, including on BP. In rodents, both a high-fiber diet and acetate supplementation improved the gut microbiome, decreasing the F/B ratio, and exhibited cardioprotective effects, including decreased BP and cardiac fibrosis [98]. Future investigations should address whether a high-fiber diet may ameliorate OSA-induced hypertension by decreasing gut dysbiosis.

Among other non-pharmacological strategies used to control BP, the role of exercise in the prevention of hypertension is well accepted. Recent evidence suggests that increased physical activity restores the richness and diversity of the gut microbiome in IH rodent models of OSA, with increases in the F/B ratio and downstream improvements in metabolic function [66]. Thus, it is possible that enhanced microbiome may mediate the antihypertensive effects of exercise in OSA.

With regard to the medical management of hypertension, several antihypertensive medications may affect microbiotal features [99]. The drop in BP caused by renal denervation was associated with a rebalance of the gut microbiota in animal models of OSA-induced hypertension [100]. Renal denervation improved microbial diversity and composition so that treated animals were not segregated from non-OSA mice. However, the effects of renal denervation were not mediated by TMAO, as no changes in this metabolite were noted.

## 6. Conclusions

**Figure 3:**
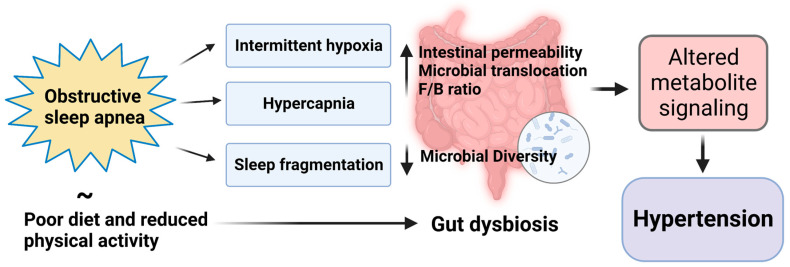
Schematic highlighting the interplay between obstructive sleep apnea and gut microbiome in altering metabolite signaling and facilitating the development of hypertension. F/B—Firmicutes-to-Bacteroidetes ratio. Created with BioRender.com.

Contemporary evidence from animal models and human studies converges to identify a contributing role of gut dysbiosis in the development and progression of hypertension in OSA (Figure 3).

OSA and its key features (i.e., intermittent hypoxia/hypercapnia and sleep fragmentation) impair intestinal function by causing epithelial damage and dysbiosis of the intestinal flora, increasing gut permeability and bacterial translocation and compromising tight junction integrity. OSA diagnosis is associated with blunted diversity in gut microbiotal communities, and a shift in composition consistent with a higher F/B ratio. Perturbations in the makeup of gut bacteria across multiple taxa, with overgrowth of pro-inflammatory bacteria and depletion of anti-inflammatory bacteria, lead to disrupted metabolite production, with increases in LPSs and decreases in SCFAs. Due to the damaged gut barrier, microbiota components and metabolites can cross the intestinal walls and spill over into target organs, resulting in systemic consequences. Consequently, gut dysbiosis, in concert with dietary factors and the brain–gut axis, presumably contributes to increases in inflammation, oxidative stress, metabolic dysregulation, and neurovascular dysfunction in OSA, ultimately promoting BP elevation and thus increasing CV risk.

Given the implications of aberrant gut microbiome for OSA-induced hypertension, restoration of intestinal microbiota communities and related functions may confer health benefits that encompass BP control. Support for this idea is provided by studies demonstrating BP-lowering effects of probiotic supplementation and high-fiber diets, among other interventions.

Future research should expand our knowledge of the interplay between OSA, microbiome and BP. Whether demographic characteristics modulate the impact of OSA on gut microbiota and microbiota-related BP increases is unknown. The contribution of specific OSA phenotypes to microbiotal alterations deserves investigation, as does the impact of gut dysbiosis on 24 h hypertension profiles. Pre- and probiotics may be assessed as adjuvant therapeutics in OSA to lower BP and alleviate CV complications by normalizing the intestinal microbiota ecosystem.

In conclusion, gut dysbiosis contributes to raise BP in OSA and may represent a novel therapeutic target to mitigate the risk of hypertension in patients with this sleep disorder.

## Data Availability

Not applicable.
